# One-shot, low-dosage intratympanic gentamicin for Ménière's disease: Clinical, posturographic and vestibular test findings

**Published:** 2014

**Authors:** Ahmad Daneshi, Hesam Jahandideh, Seyed Behzad Pousti, Shabahang Mohammadi

**Affiliations:** 1 Department and Research Center of Otolaryngology-Head and Neck Surgery, Hazrat-e-Rasool Akram Hospital, Iran University of Medical Sciences, Tehran, Iran; 2 Department of Otolaryngology-Head and Neck Surgery, School of Medicine, Iran University of Medical Sciences, Tehran, Iran; 3 Department and Research Center of Otolaryngology-Head and Neck Surgery, School of Medicine, Iran University of Medical Sciences, Tehran, Iran

**Keywords:** Ménière's Disease, Gentamicin, Vestibular Evoked Myogenic Potentials, Dynamic Posturography

## Abstract

**Background:**

Ménière's disease has been remained as a difficult therapeutic challenge. The present study aimed to determine the effects of one-shot low-dosage intratympanic gentamicin on vertigo control, auditory outcomes and findings of computerized dynamic posturography and vestibular evoked myogenic potentials in patients with unilateral Ménière's disease.

**Methods:**

In a prospective clinical study, 30 patients with unilateral Ménière's disease were treated with one-shot intratympanic injection of 20 milligrams gentamicin. Main outcome measures included clinical, audiometric, postural and vestibular outcomes evaluated 1 and 9 months after the treatment.

**Results:**

Mean vertigo attacks frequency, pure tone average threshold and functional level scale significantly decreased after the treatment (P < 0.05). Effective vertigo control (class A and B) obtained in 95.8% of the patients. In total, 75% of patients reported decrease in both aural fullness and tinnitus. Vestibular evoked myogenic potentials became absent in all the patients but four of them. Posturographic scores were improved after the treatment.

**Conclusion:**

One-shot low-dosage gentamicin was effective in controlling vertigo attacks in Ménière's disease and has useful effects on aural fullness and tinnitus of patients as well. Postural and vestibular tests only have adjunctive role for monitoring therapeutic responses in intratympanic gentamicin-therapy.

## Introduction

Ménière's disease is a clinical disorder defined as the idiopathic syndrome of endolymphatic hydrops.^1^ It is an obvious finding that the Ménière's patients rated their quality of life (QoL) significantly worse in both the physical and psychosocial dimensions than the normal healthy subjects. Previous studies have shown that vertigo has more impact on the physical aspects, whereas tinnitus and hearing loss seemed to influence the psychosocial and emotional aspects more than the physical aspects.^2^ There is no cure for Ménière's disease and interventions do not eliminate the underlying cause of it.

In recent decades, intratympanic gentamicin administration for treatment of Ménière's disease has gained widespread popularity and has demonstrated its clinical effectiveness in the control of intractable vertigo associated with Ménière's disease in a variety of clinical studies.^3, 4^ Different methods of administration with gentamicin include multiple daily dosing, weekly administration for four total treatments, low-dose therapy consisting one to two injections with repeating treatment only for recurrent vertigo symptoms, continuous micro-catheter delivery and titration of therapy to the onset of inner ear disturbance. Although numerous articles have been published using each of these techniques, to date there is no agreement between the otolaryngologists regarding which technique offers the greatest amount of vertigo control with the lowest rate of complications.^5^ According to the concept of partial vestibular ablation, only reduction in vestibular function may be enough in most patients to control the vestibular symptoms of the disease.^5^ However, a recent meta-analysis have shown that the low-dosage method, in contrast, trends toward worse effective vertigo control than the other methods.^3^

Newer techniques such as the computerized dynamic posturography (CDP) have facilitate objective evaluation of patients with balance disorders. This technique allows analysis of the information supplied by the three sensory systems (i.e. visual, vestibular and proprioceptive) which contribute to the maintenance of balance. CDP has been shown to be a cost-effective and useful technique for the characterization and monitoring of patients with balance disorders.^6^

Brief, loud, monaural clicks or tone bursts produce a large, short-latency, inhibitory potential in the tonically contracted ipsilateral sternocleidomastoid muscle. These potentials have come to be known as vestibular evoked myogenic potentials (VEMPs). There is good evidence that the VEMPs is a vestibulocollic reflex; thus, the VEMPs can be used as a test of otolith organ and peripheral vestibular function.^7^

## Materials and Methods

In a prospective clinical study, 34 patients with unilateral definite Ménière's disease according to the definition of the American Academy of Otolaryngology-Head and Neck Surgery (AAO-HNS)^8^ were included in the study from January 2009 to February 2011. Criteria for offering intratympanic gentamicin as a treatment option included intractable vertigo despite lifestyle modification and drug-therapy (2 g per day sodium diet, diuretics and betahistine) for at least 1 year, no symptoms suggestive of auditory or vestibular disease in the contralateral ear, and serviceable hearing in the contralateral ear. No patient had history of previous ear surgery, neurologic disorder or aminoglycoside sensitivity. At the beginning of study, after explanation of treatment efficacy and probability of complications including dead ear and balance problems such as “curative vertigo”,^9^ each patient gave written informed consent for treatment with intratympanic gentamicin. Pretreatment evaluation included a complete neuro-otologic evaluation, magnetic resonance imaging (MRI), pure tone average (PTA) measurement and impedance audiometry, CDP and VEMPs. Moreover, frequency of vertigo, functional level and severity of aural fullness and tinnitus were recorded. An arbitrary scoring system (0 = none, 1 = mild, 2 = moderate, 3 = severe) used for aural fullness and tinnitus grading.

The saccular function was tested by the measurement of the VEMPs. Electromyography (EMG) activity was recorded with a Micromed electromyogram (EMG) system from the middle third of each sternocleidomastoid, i.e. a reference electrode on the mastoid and a ground electrode on the forehead. During the measurements, the test subject was supine and was required to raise and hold his/her head to tension the muscle. The acoustic stimuli (tone burst; 500 Hz; 115 dB peSPL; 9/s) were presented via a headphone. Potentials were amplified and bandpass filtered (20–2500 Hz). Recording window was 50 ms. Two runs from each side were obtained. In each trace, we considered the latency and amplitude of the two positive/negative waves (p13–n23). Amplitude ratios between the two ears were calculated as previously described.^10^ Amplitudes and latencies recorded from the affected ear were compared with those obtained from the healthy contralateral ear and a group of 20 healthy control subjects.

CDP performed with EquiTest (Neurocom International, Clackamas, Oregon, USA) equipment using standard EquiTest protocol for testing. While viewing a visual surround the composite equilibrium score was recorded for analysis that was automatically computed by the standard algorithm in the computer software. The equilibrium scores can range from 0 to 100 with 100 indicating no movement on the posture platform. Each one of these six conditions was repeated three times in a standard fashion. These sensory conditions were:^6, 11^ 1. Immobile surface, immobile visual surround, eyes open (assessing visual, vestibular and proprioceptive systems); 2. Immobile surface, eyes closed (assessing vestibular and proprioceptive systems); 3. Immobile surface, mobile visual surround, eyes open (assessing vestibular and proprioceptive systems); 4. Mobile surface, immobile visual surround, eyes open (assessing visual and vestibular systems); 5. Mobile surface, eyes closed (assessing vestibular system); 6. Mobile surface, mobile visual surround, eyes open (assessing vestibular system). In total, 18 scores were obtained, three for each of the six conditions. The arithmetic mean of the three scores of each condition was used for the statistical analysis. To objectively assess the vestibular part of the balance system separately, the following parameters were considered in the data analysis: condition 5 score, condition 6 score, composite score, and vestibular ratio (VEST). VEST ([condition 5 score]/ [condition 1 score] *100) is a measure of the patient's ability to use vestibular information for maintenance of balance.^6^

Ten minutes before the injection, the tympanic membrane was anesthetized with topical tetracaine drop. During the procedure, each patient lay supine and the tympanic membrane of the involved ear was visualized under the operating microscope with the head turned 45 degree to the opposite side to prevent leakage of the solution through the Eustachian tube and to allow for adequate contact of the drug with round window membrane. The 0.5 mL of the stock gentamicin solution (40 mg/ml) was drawn into a tuberculin syringe and with A 25-gauge, 3.5" slightly bent spinal needle was injected posteroinferiorly. After the injection, the patient maintained this head position for at least 30 minutes and was told to avoid swallowing to prevent any opening of the Eustachian tube. Few patients experienced some burning pain lasted seconds and resolved immediately.

Patients were asked to consider water precautions for two weeks and return for initial follow-up after four weeks. In this follow-up session, the frequency of vertigo, functional level, aural fullness and tinnitus changes and severity of these symptoms were recorded and audiometry, CDP and VEMPs were repeated. Patients were also asked to return about seven months later for the second follow-up.

We conducted statistical analysis using SPSS Predictive Analytics Software Statistics (PASW) for Windows 18.0 (SPSS Inc., Chicago, IL, USA). Comparisons of frequency of vertigo attacks, audiometric data and functional level scale before and after the treatment were made with general linear model repeated measures tests. A nonparametric comparison of numbers of patients in different outcome groups was made with the *χ* ^2^ test.

## Results

**Figure 1:**
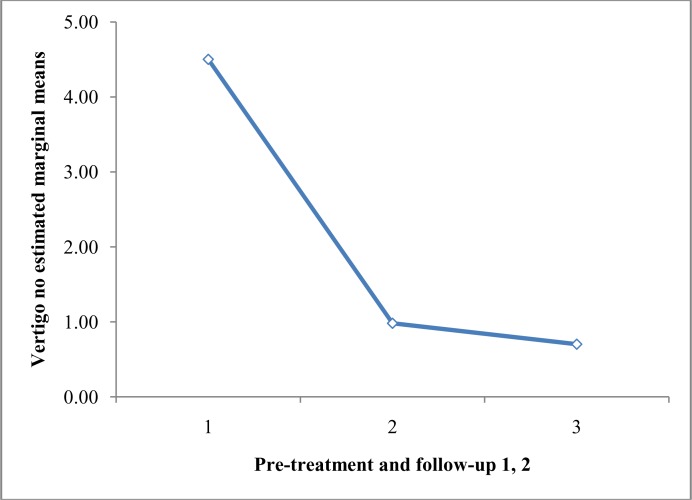
Frequency of vertigo attacks before and after the treatment

Demographic data, frequency of vertigo attacks, audiometric data and functional level scale of the patients are shown in Table 1 (according to reporting style recommended by AAO-HNS). Initial follow-up and late follow-up were done after (1.32 ± 0.53) and (9.92 ± 3.99) months, respectively. Mean affliction time of patients were (41.27 ± 23.38) months. About 93% of the patients mentioned curative vertigo at the first week after gentamicin injection. Mean vertigo attacks frequency (Figure 1), PTA thresholds, speech recognition threshold (data not shown) and functional level scale decreased significantly after treatment (P < 0.05). Changes in SDS and 4 and 8 KHz average threshold were not statistically significant. Since the follow-up did not extend as recommended by the Committee on Hearing and Equilibrium, the frequency of vertigo attacks was calculated based on the last 6 months of follow-up. Among 24 patients with late follow-up, 14 patients (58.3%) were in class A (remission), 9 patients (37.5%) in class B (substantial control) and 1 patient (4.2%) in class C. patients with recurring vertigo attacks stated that intensity of each attack had been reduced and their attacks were more tolerable.

**Table 1:** Demographic data, frequency of vertigo attacks, audiometric data and functional level scale of the patients before and after the treatment

					Baseline					Initial follow-up					Late follow-up				
							
Pt No	Age	Sex	Side	Stage	FV	PTA	SDS	4,8 KHz	FL	FV	PTA	SDS	4,8 KHz	FL	FV	PTA	SDS	4,8 KHz	FL
1	24	M	R	2	5	35	100	15	5	3	15	100	25	3	0	5	100	20	2
2	26	F	R	3	5	60	60	60	3	1	30	100	10	3	1	45	88	35	3
3	37	M	R	3	3	50	72	40	4	1	40	92	45	2	1	40	92	40	2
4	63	M	L	3	5	55	28	50	3	2	50	20	40	5	--	--	--	--	--
5	57	M	L	3	4	45	100	70	3	2	40	100	75	3	1	35	100	75	3
6	72	F	R	3	6	65	--	75	4	2	65	--	60	2	1	70	--	60	2
7	51	F	L	2	5	35	100	15	3	0	40	100	45	3	0	45	92	35	2
8	42	F	L	2	7	40	100	10	3	1	30	100	40	2	0	10	100	30	2
9	38	F	L	2	3	40	100	35	4	0	35	96	40	3	--	--	--	--	--
10	25	M	R	1	2	15	100	5	4	0	10	100	50	2	0	10	100	40	2
11	33	F	L	3	2	60	36	50	4	0	55	40	65	2	0	30	96	50	2
12	42	F	R	3	3	50	80	60	5	1	40	92	60	4	0	25	96	50	1
13	54	F	R	3	4	55	88	55	4	0	20	100	45	1	0	30	80	45	1
14	21	M	R	1	5	10	100	10	3	0	20	100	5	2	0	10	100	0	2
15	52	F	L	4	7	95	--	80	3	1	85	--	80	3	1	90	--	80	3
16	62	M	L	2	4	40	100	50	2	0	55	80	55	2	0	40	100	50	2
17	38	M	L	3	3	60	96	65	3	0	50	56	65	3	1	55	76	65	2
18	26	M	R	1	3	25	100	25	3	--	--	--	--	--	0	15	100	40	1
19	39	F	R	2	4	35	100	45	2	1	30	92	55	2	1	20	92	40	2
20	38	F	L	3	2	50	92	55	4	0	55	44	70	3	1	55	48	65	2
21	53	M	R	3	7	45	84	65	3	0	70	60	75	3	0	65	32	85	2
22	57	F	R	1	3	20	100	40	2	0	10	100	45	2	0	15	100	45	2
23	36	M	L	3	3	55	100	55	3	0	50	100	60	2	0	45	100	50	2
24	38	F	L	3	6	55	52	60	4	1	35	96	60	3	1	40	88	45	2
25	47	M	L	3	7	50	80	65	5	2	55	68	70	4	2	60	36	65	3
26	53	M	L	3	3	70	88	70	4	1	45	88	70	3	--	--	--	--	--
27	70	M	R	2	3	30	96	75	3	--	--	--	--	--	0	100	--	110	2
28	44	M	L	3	5	45	100	55	3	1	40	80	50	3	--	--	--	--	--
29	54	F	L	2	4	30	100	50	3	0	20	100	40	2	--	--	--	--	--
30	54	F	L	3	4	70	--	85	3	0	65	.	85	2	--	--	--	--	--
Mean	44.87			2.50	4.23	46.33	87.11	49.67	3.40	0.71	41.25	84.16	53.04	2.64	0.46	39.79	86.48	50.83	2.04
SD	13.64			0.78	1.55	17.86	20.50	21.97	0.81	0.85	18.59	22.91	19.21	0.83	0.59	25.17	21.30	22.68	0.55
P										0.00	0.01	0.84	0.70	.00	.00	0.01	0.84	0.70	.00

FV = Frequency of vertigo; PTA = Pure tone audiogram; SDS = Speech discrimination score; FL = Functional level

Regarding aura fullness and tinnitus of patients, data are shown in Table 2. Posturographic data are shown in Table 3. Although overall scores increased after treatment this differences did not reach statistical significance.

**Table 2:** Aural fullness and tinnitus changes

	Pre-treatmentScore (SD)	Initial follow-upchange(n = 28)	Initial follow-up Score (SD)	Late follow-up changes (n = 24)	Late follow-up Score (SD)
Aural fullness	2.27 (0.74)	12 D/1 I/15 NC	1.71 (0.90) P = 0.00	18 D/6 NC	1.25 P = 0.00
Tinnitus	2.53 (0.68)	14 D/1 I/13 NC	1.86 (0.93) P ≤ 0.001	18 D/2 I/4 NC	1.63 (0.82) P ≤ 0.01

D = Decreased, I = Increased, NC = Not changed

**Table 3:** Posturographic data

	Baseline	Initial follow-up	Late follow-up
Condition 5 score	57.83	61.71	61.79
Condition 6 score	53.32	57.14	53.93
Composite score	74.13	76.36	75.29
VEST	60.79	65.33	65.54

VEST = Vestibular ratio

Before the treatment, VEMPs were positive in 23 out of 30 affected ears and 29 out of 30 healthy ears. Mean latency for p13 and n23 waves in affected ear were 14.68 ms and 19.20 ms, respectively. In healthy side, these were 13.74 ms and 18.55 ms, respectively. In 20 healthy control ears, mean latency for p13 and n23 waves were 12.19 ms and 17.48 ms, respectively. Mean amplitude of affected ear, healthy ear and healthy controls were 25.07, 27.42 and 43.96 µv, respectively. There was not any significant difference between the affected ear and healthy ear and normal subjects in latency times, amplitudes and amplitude ratio. In initial and late follow-up, VEMPs did not disappear in 4 patients and became absent in remaining.

## Discussion

According to the current medical literature, intratympanic therapy has emerged as a standard mode of therapy to decrease vertigo associated with Ménière's disease.^3^ Moreover some studies suggest that gentamicin definitely improves quality of life (QoL) in patients with Ménière's disease and should be the first line of treatment if medical management fails.^12^ Some authors propose that only complete ablation of vestibular function with gentamicin therapy can effectively treat Ménière's disease while another group believes that only reduction in vestibular function may be enough in most patients to control the vestibular symptoms of the disease.^5^ In a very interesting meta-analysis to establish safe dosing protocols of intratympanic gentamicin for treatment of patients with Ménière's disease, Salt et al. have used a computer simulation program which calculates drug dispersal in inner ear fluids. Using this model of gentamicin dispersal, they have estimated perilymph and cochlear gentamicin levels following specific application protocols in humans. They have shown that drug levels resulting from single, “one-shot” injections were typically lower than those from repeated or continuous application protocols. They have concluded that one-shot application protocols produce gentamicin doses in the cochlea that have minimal risk to hearing and repeated or continuous application protocols result in higher doses that in some cases damage hearing.^13^ Interestingly, in another meta-analysis, Chia et al. concluded that low doses gentamicin resulted in lowest rate of vertigo control (86.8%) compare to other methods while hearing loss is comparable to them.^3^

However in our study, using one-shot low-dosage gentamicin led to complete vertigo cessation (class A) in 58.3% of the patients and effective vertigo control (class A and B) in 95.8%. Although complete cessation of vertigo attack (class A) in our study was lower than what has been reported in related studies of low-dose gentamicin, our rate of effective vertigo control was comparable or higher.^5, 14–17^ It also should be mentioned that most of these studies have used higher doses or repeated injections. Indeed, many of repeatedly cited studies about low-dose or single-dose gentamicin are not actually merit it. Although Minor's study usually cited as prototype of single-dose therapy, only 17% of the patients received single injection.^14^ Furthermore, in a second group of Abou-Halawa and Poe study,^18^ only 33% of the patients and in Lange et al. study,^8^ only 53% of the patients received single dose therapy.

Although mean change PTA was favored regarding better thresholds, 20.83% of our patients had worse PTA according to significant change of 10 dB or more defined by AAO-HNS. Moreover one of our patients developed profound hearing loss following single low-dose therapy with gentamicin. The response to gentamicin is notoriously unpredictable and profound hearing loss after a single injection is possible. Idiosyncratic response resulting from a genetic enzymatic defect may be the underlying cause.^18^ According to Chia et al., meta-analysis toxic effects of intratympanic administration of gentamicin on hearing and word recognition were found to be neither statistically significant nor clinically important.^3^ However, interpretation of these results is complicated because of natural course of Ménière's disease itself. Some studies also have shown that delayed onset or late hearing deterioration in approximately one third of patients treated with intratympanic gentamicin. However, using historical control, researchers found this hearing loss similar to that seen in the natural progression of Ménière's disease.^18^ Review of the PTA before and after the treatment in similar studies also have shown wide range of responses, from a decrease of 30 dB to an improvement of 58 dB.^19^

In the second follow-up, 75% of our patients reported decrease in both aural fullness and tinnitus. Similar findings regarding fullness have been reported previously,^20^ but our results about tinnitus decrease were striking. Some investigators have proposed that gentamicin exerts its effects primarily not only on the sensory hair cells but also on the destruction of the dark cells within the labyrinth that are responsible for endolymph production. Thus, the effect of gentamicin should be due to not only destruction of vestibular function but also relieving endolymphatic hydrops.^16^

Despite the well-documented limitations of the caloric test, caloric responses persevere as the gold standard among clinicians for the diagnosis and management of vestibular disorders, recent studies challenge this classic dictum though.^10^ Moreover, studies have shown that with regard to determining the efficacy of intratympanic gentamicin treatment in patients with Ménière's disease, the reliability of testing for VEMPs is comparable to that of caloric tests. Compared with caloric tests, VEMPs measurements are more comfortable and take less time.^17^ Complete loss of vestibular function as manifested by absent caloric response to need not be pursued as a therapeutic end point in intratympanic gentamicin therapy, as it is not required for vertigo control in Ménière's disease.^17^ VEMPs testing is a useful adjunct to other studies because it can reveal the saccular dysfunction. After the cochlea, the saccule is the second most common site of hydropic changes in temporal bones of patients with Ménière's disease.^21^

Comparison between latency of p13 and n23 waves and amplitude of p13-n23 complex between affected ear and health side and healthy controls showed increased latency and diminished amplitude, this finding is concordant with others findings^15^ but our amplitude values while similar with some studies^22^ are somewhat different with others.^10, 23^ Confounds potentially affect VEMPs responses and comparisons between investigations show multiple differences in methodology such as maximum output levels, repetition rate, stimulus duration, and method of sternocleidomastoid muscle activation and monitoring.^22^ It seems that now with more widespread use of VEMPs, this test needs developing standard protocols for conduction and reporting. Before the treatment, VEMPs were absent in 23.33% of our patients, result that are compatible with others findings.^10, 15, 24–26^ However, VEMPs disappeared in only 81% of our patients after the therapy. In another similar study of low-dose gentamicin, VEMPs also disappeared in 75% of patients but this is somewhat less than other studies with 100% disappearance.^10, 15^ Interestingly, patients whose their VEMPs did not disappeared, had no recurrent vertigo attack and this finding weakens the role of VEMPs as a predictor of post treatment sixth-month patient status and vertigo control as suggested in some studies.^23^ Whether partial vestibular ablation concept^5^ again may explain this finding is not clear.

Studies have found that CDP documents the presence of vestibular abnormalities in a significant number of subjects with normal calorics and other peripheral vestibular disorders. CDP complements vestibulo-ocular reflex tests of vestibular function.^10^ Although did not reach statistical significance, our results show that intratympanic gentamicin in patients affected by unilateral Ménière's disease improves CDP scores both as early as 4 weeks and also in later follow-up. Other studies also demonstrated that after gentamicin therapy postural stability improves.^10^ CDP can act only as adjunctive test to objectively demonstrate clinical improvement.

As our study limitations, this study is non-blinded and does not contain a control group but it is not plausible to withhold an effective treatment from a group of patients. We could not follow our patients for long time as recommended by AAO-HNS criteria because of multiple local factors including most of our patients were referred from other cities. Previous studies have also shown that the adherence of researchers to the AAO-HNS criteria was partial and was found to be adequate in only 50% of the publications.^27^ Further investigation about role of even lower doses of gentamicin on quality of life, vertigo control and auditory outcomes and role of VEMPs in the treatment process are warranted.

## Conclusion

One-shot low-dosage gentamicin is completely effective on controlling vertigo attacks in Ménière's disease and has useful effects on the aural fullness and tinnitus of patients as well. However, even doses as low as 20 mg gentamicin can cause hearing loss. VEMPs and CDP may have only adjunctive role in monitoring therapeutic responses in intratympanic gentamicin therapy.
